# Research on Key Techniques of Insect Flapping Onset Control Based on Electrical Stimulation

**DOI:** 10.3390/s20010239

**Published:** 2019-12-31

**Authors:** Yu Feng, Bo Yang, Yongchang Jiang, Xiang Zheng

**Affiliations:** 1 School of Instrument Science and Engineering, Southeast University, Nanjing 210096, China; 2 Key Laboratory of Micro-Inertial Instrument and Advanced Navigation Technology, Ministry of Education, Nanjing 210096, China

**Keywords:** electrical stimulation, micro-control system, flapping onset control, dorsal longitudinal muscle

## Abstract

In this paper, an insect flapping onset control method based on electrical stimulation is proposed. The beetle (*Allomyrina dithotomus*, Coleoptera) is employed for the research carrier, and it’s left and right longitudinal muscles are electrically stimulated to control the flapping onset behavior. The control principle of insect flapping onset utilizing electrical stimulation is analyzed firstly followed by the movement function of the dorsal longitudinal muscle. Subsequently, a micro-control system, which is composed of a PC controller, coordinator and electronic backpack, is designed to realize the wireless control of beetle movements. Finally, the verification experiment is implemented to verify the effectiveness of dorsal longitudinal muscle stimulation with respect to the beetle flapping onset, whereas the comparative experiment emphasizes on determining optimal simulating parameters. The experimental results demonstrate that when the period, duty ratio, number of and amplitude of pulses stimulation signal are assigned to 5 ms, 20%, 90 and 3.3 V respectively, the beetle flapping onset can be controlled with an average response time of 1.69 s. Simultaneously, the optimization of duty ratio from 20% to 40%, and the number of pulses from 90 to 100, is proved to the best parameter configuration.

## 1. Introduction

The concept of incorporating microcomputers with living insects, which takes advantage of the great athletic ability of insects and control ability of microcontrollers has been conceived by numerous research groups as early as the 1990s. With the rapid development of low-power microcontrollers and radio technology in recent years, wireless control, feedback control, intelligent algorithms and many other techniques have emerged for the insect–computer hybrid robot employment. The insect-computer hybrid robot inherits the excellent stability, agility and maneuverability from the insect’s own motion systems, and feature low energy consumption, low cost, high reliability and replicability simultaneously, thus satisfying the application requirements in harsh environments. The insect-computer hybrid robot has a wide application prospect in the field of military strategy, national defense security and insect scientific research. It is also expected to implement some special work for humans, such as searching for disaster survivors, carrying out military reconnaissance, and so on [1,2,3].

C.J Sanchez in the Texas A&M University, the United States, who developed an electronic backpack for the electrical stimulation of the left and right of the thoracic ganglion in the cockroaches, successfully implemented the left and right steering of the cockroaches in 2015 [4]. A micro-light polyimide flexible waveguide platform was proposed in Draper Laboratory for the flight steering of dragonfly in a free state [5]. In 2009, the take-off, stop and turn of Manduca sexta were successfully controlled at Cornell University, where an electronic backpack was developed for electrically stimulating the nerves and muscles of Manduca sexta [6]. Microfabricated flexible neural probes (FNPs) were developed in the Massachusetts Institute of Technology in 2011, which form a bi-directional electrical link to the moth Manduca sexta. The researchers used FNPs to induce moths to turn left and right [7,8]. K.Mann in Washington University integrated sensors, computers and communications functions into bees, using insects instead of drones to create a removable animal networking platform in 2018 [9]. The left and right optic nerve leaves of beetles are successfully stimulated by an electronic backpack for the takeoff control at the University of California at Berkeley in 2009 [10,11,12]. H.Sato in Nanyang Technological University found that the contraction degree of beetle 3Ax muscle was graded with the change of stimulation frequency in 2014 [13]. On this basis, a feedback PD controller was designed in 2018 to control the horizontal flight of beetle 3Ax muscle [14].

In this paper, we propose an insect flapping onset control method based on electrical stimulation. In order to meet the requirements of large volume and certain load capacity of biological carriers, we select beetles as the research objects. In Section 2, a brief description of the control principle of beetle flapping onset and a subsequent micro-control system scheme is presented. Then, the demonstration of the results of validation and comparison experiments are exhibited in Section 3. Concluding remarks are finally given in the last section. The purpose of the research is to develop the insect-computer hybrid robot for beetle flight behavior controlling. The control of insect flight behavior generally includes the initiation and stop of flight action and the control of flight direction. Controlling the wings vibration is the first step to control beetle flight behavior, which plays an important role. Therefore, this paper focuses on how to effectively control the wings vibration through electrical stimulation.

## 2. Principle and Method

### 2.1. Structure Design

As well known, the muscles contraction and relaxation of beetle are controlled by specific nervous systems. The nervous systems directly affecting the beetle movement behavior include the brain, ventral nerve cord and peripheral nervous system. The movement muscle system which directly controls the flight and swing of beetles is comprised of basilar muscle, dorsal longitudinal muscle, dorsal abdominal muscle, etc.

**Figure 1:**
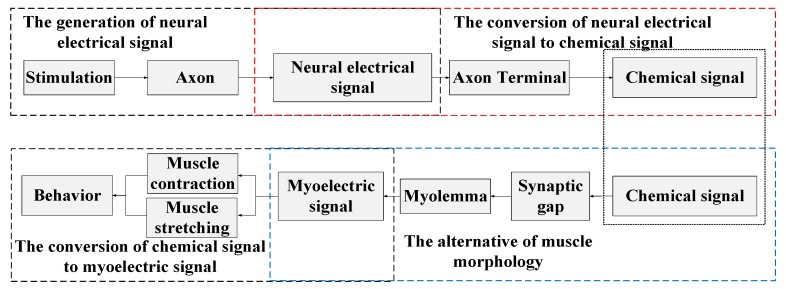
The movement mechanism of beetles.

The complete control process of nervous systems over muscles contraction and relaxation can be summarized as four steps, including the generation of neural electrical signal, the conversion of neural electrical signal to chemical signal, the conversion of the chemical signal to myoelectric signal and the alternative of muscle morphology, as shown in Figure 1. Neural electrical signals reflecting external stimulation are generated due to the inequality of the Na ions flow between the two sides of the axon membrane. The imbalance of ions can further yield an action potential difference and thus form a voltage signal. The generated neural electrical signals are conducted alongside the axon and then fed into nerve–muscle joint mechanism which is capable of converting them into chemical signals. The nerve synapse in this mechanism is responsible for transforming action potential into neurotransmitter acetylcholine (chemical signals) and transmitting it to the muscle cross the synaptic gaps. Afterward, the arrival of preceding neurotransmitters (chemical signals) to the muscle membrane yields the flow of the Na/K ions inside and outside it, thus forming a similar action potential which is called myoelectric signal. Subsequently, the entry of myoelectric signal into muscle tissue triggers a series of biological reactions between actin and myosin, and induces the muscle contraction and relaxation. In light of this, the microscopic muscle actions finally result in macroscopic beetle behaviors [15].

**Figure 2:**
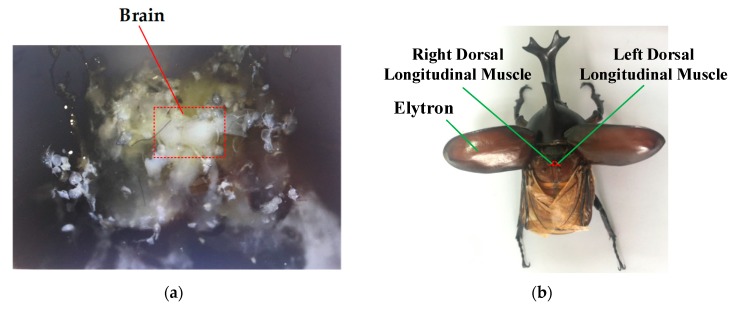
Beetle biological structure for flapping onset movement control system. (**a**) Movement control nerve and brain of beetle; (**b**) the left and right longitudinal dorsal muscle of beetle for implant sites.

The brain and abdomen of beetle are dissected by anatomical techniques, illustrated in Figure 2. The dorsal longitudinal muscle and dorsal ventral muscle of beetle are a pair of antagonistic flying muscles, which are located in a side domain and mid-domain of the beetle’s thorax, respectively, and are responsible for the upper and lower flapping of the wings. To initiate wings flapping, either or both the dorsal ventral muscle and the dorsal longitudinal muscle should be stimulated. The dorsal ventral muscle is enclosed by the cuticle. When the dorsal ventral muscle is used as the stimulation site, part of that cuticle is destroyed by the electrode implantation, reducing the power output of the dorsal ventral muscle. It will affect the flight ability of the beetle. Therefore, we choose the dorsal longitudinal muscle as the stimulation site and can control beetle flapping onset successfully. In addition, Choo et al. successfully controlled the wings flapping of beetle (*Mecynorrhina torquata*, Coleoptera) by applying 3 V electrical pulses signals at 100 Hz to the dorsal longitudinal muscle [16].

The wings of beetle swing resemble to a tuning fork. When the wings swing downward, the dorsal longitudinal muscle contract and the dorsal ventral muscles stretch; when the wings swing upward, the dorsal longitudinal muscle stretch and the dorsal ventral muscle contract. Two groups of muscles contract and stretch alternately, which pull wings directly up and down and distort the thorax, finally causing beetles to flapping onset. Considering that the significant power spectrum of myoelectric signals ranges from approximately 20~500 Hz [17], we adopt the electric stimulation pulses sequence of 5 to 35 ms (28.6~200 Hz) to directly imitate the beetle myoelectric signal and exert on the dorsal longitudinal muscle so that dorsal longitudinal muscle can contract and stretch, therefore realizing flapping onset. Because the electrical stimulation pulses simulate the electrical signal exerted by the nervous system on the dorsal longitudinal muscle, which plays the role of inducing the beetle to flapping. Therefore, there is no need to induce the flapping behavior of the wings by directly tuning the stimulation signal to the natural vibration frequency of the wings.

### 2.2. Design of Micro-Control System

#### 2.2.1. Hardware Design of Micro-Control System

**Figure 3:**
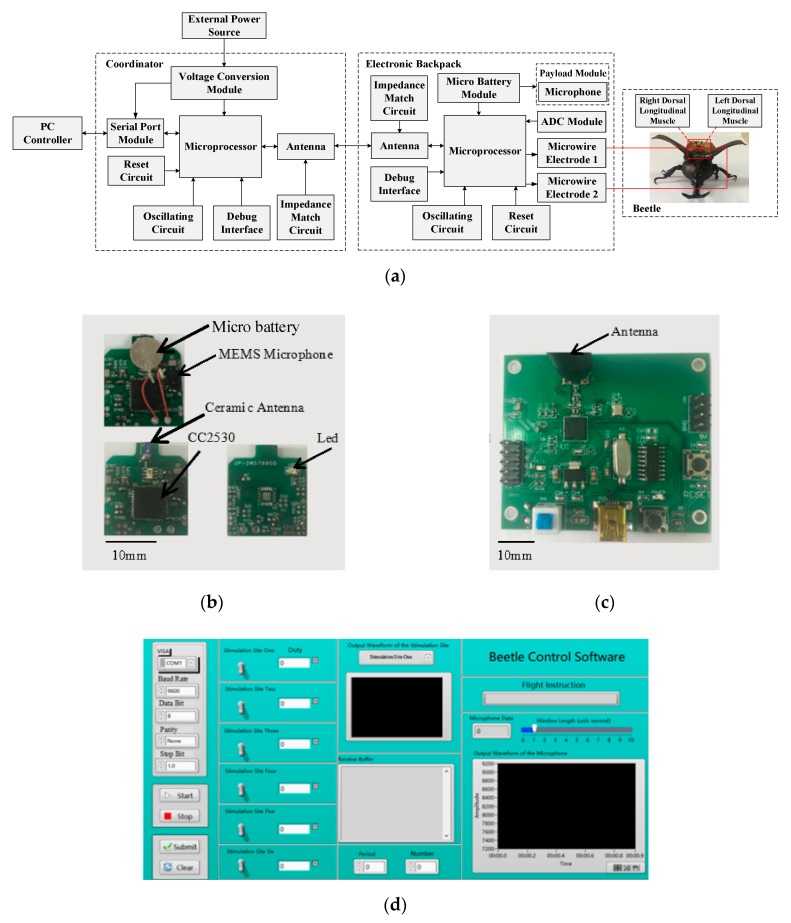
Micro-control system. (**a**) Schematic of the hardware design of micro-control system; (**b**) physical map of electronic backpack (16 mm × 16 mm); (**c**) physical map of coordinator; (**d**) PC controller interface.

According to the control requirements of the beetle flapping onset movement, the hardware of the micro-control system which consists of the coordination and the electronic backpack is designed in Figure 3.

The coordination is composed of a microprocessor (CC2530 chip of TI), a peripheral circuit (a reset circuit, an oscillating circuit and a debug interface), an antenna with the impedance matching circuit, a serial port module and a voltage conversion module. The main functions of coordination are communication and signal transmission and networking. The wireless local area network is established to achieve resource sharing and communication by coordination, which can provide data transmission between the PC controller and the electronic backpack. The first function of the microprocessor integrated chip is to receive stimulation command from the PC controller and deliver it to the electronic backpack. Another function is to receive the microphone information from the electronic backpack and deliver it to the PC controller. The serial port module is used for the two-way communication between coordination and PC controller. The voltage conversion module transforms the external power supply module into the appropriate voltage for the microprocessor. The antenna is used for signal reception and transmission between coordination and electronic backpack by remote wireless communication. The impedance matching circuit of the antenna applies to optimize signal transmission distance and quality.

The electronic backpack consists of a microprocessor (CC2530 chip of TI), a peripheral circuit (a reset circuit, an oscillating circuit, a debug interface), a ceramic antenna (YAGEO3216) with the impedance matching circuit, a micro battery module, a micro-sensor module (microphone), an ADC module and a microwire electrode module. The main functions of the electronic backpack module are communication transmission, beetle behavior control and payload signal processing. The communication transmission is used to receive the stimulation command issued by the coordination, and meanwhile to deliver the data collected and processed by sensor back to the coordination. According to the corresponding electrical stimulation signal generated by stimulation command, the beetle behavior control is to electrically activate the stimulation sites of beetles’ left and right dorsal longitudinal muscles by the microwire electrodes. The payload signal processing is implemented by the microsensor module for monitoring and recording the wings vibration information. The microprocessor is used to generate and output electrical stimulation signals on the basis of stimulation command and transmit the wings vibration information of the beetles to the coordination. The MEMS analog microphone is adapted to collect the vibration information of wings. The ADC module converts the analog signal collected into the digital signal by the microphone. The rechargeable lithium micro battery is arranged to providing power for the entire backpack.

#### 2.2.2. Control Algorithm for Micro-Control System

**Figure 4:**
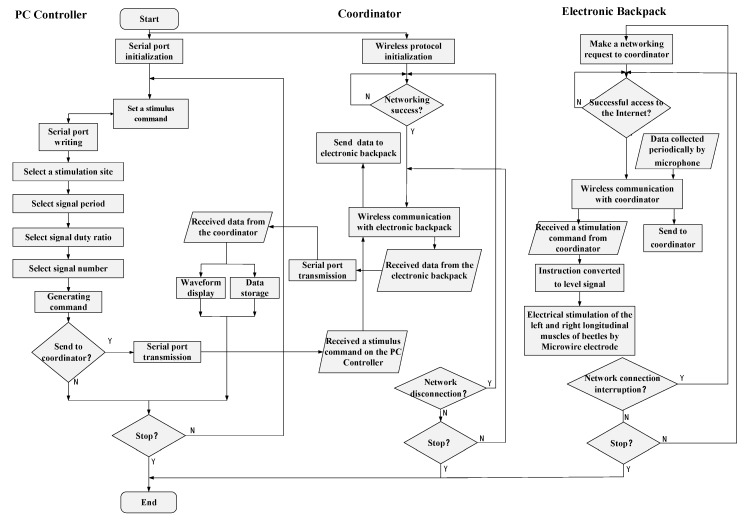
Control algorithm for the micro-control system.

In order to realize beetle flapping onset control, we designed the control algorithm by combined the above hardware equipment, shown in Figure 4. The control algorithm consists of the PC controller algorithm, the coordination algorithm, and the electronic backpack algorithm, corresponding to the PC controller, the coordination and the electronic backpack, respectively.

The PC controller is designed by LabVIEW software. The main functions are the generation of stimulation commands, signal reception and delivery, and information processing. The algorithm first initializes the serial port, then determines whether it needs to send a stimulation command. When the stimulation command is released, the serial port is set to the write state, then the corresponding stimulation command is configured by selecting the stimulation sites, the signal pulses frequency, the number and the duty ratio, which is delivered through the serial port channel. When there is no need to send a command, the serial interface keeps the reading state all the time. The information sent back by the coordination is continuously read through the serial port transmission, meanwhile, the waveform drawing and data storage are performed on the information.

The main function of the coordination algorithm is to establish the wireless local area network, implement the forwarding of the stimulation command and the reading of wings vibration information. The algorithm first initializes the serial port protocol and the wireless communication protocol. When the stimulation command is delivered to the serial port of the microprocessor, the serial port is set to the reading state so as to read and store the stimulation commands. When there is no command to be received, the serial interface is set to the writing state, and the information read and cached by the wireless communication is delivered to the PC controller. Meanwhile, the wireless local area network starts to build after the initialization of the wireless communication protocol. When the network is successfully established, the serial port cache instruction starts to be delivered, meanwhile, the wings vibration information sent from the electronic backpack is read and cached.

The main function of the electronic backpack algorithm is to execute the stimulation command, and, meanwhile, collect and process the wings vibration information when the wings are flapping, and send it to the coordination through the wireless communication protocol. The algorithm first initializes the wireless protocol and then starts to deliver the network access request to the communication coordinator to join the wireless network. When the network access is successful, it starts to receive the stimulation command issued by the communication coordinator, and read the wings vibration information collected by the microphone. The received stimulation command generates a stimulation signal through a signal generation algorithm. Then the stimulation signal passes through a specific I/O port is delivered to the microwire electrode. Simultaneously the wings vibration information is read by the microphone and processed by the ADC module, and then delivered to the communication coordinator through the wireless communication.

## 3. Experiments and Results

### 3.1. Experimental Preparation

**Figure 5:**
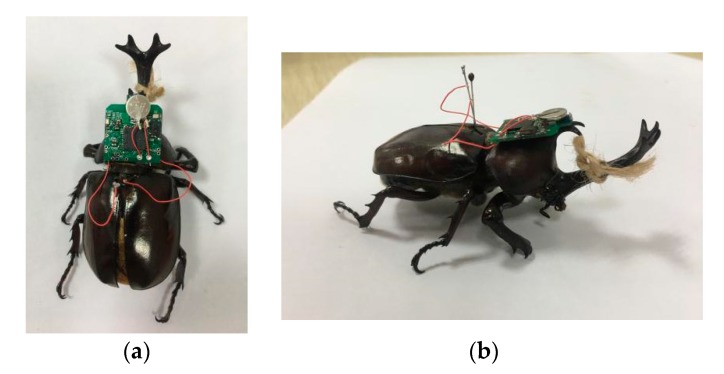
Photograph of a beetle carrying an electronic backpack. (**a**) The top view of the beetle; (**b**) the side view of the beetle.

The beetles (*Allomyrina dithotomus*, body length: 65 ± 10 mm, weight: 72 ± 2 g) used in the experiment are fed jelly every two days in a professional reptile box (32 cm × 22 cm × 22 cm). Before each experiment, the flying ability of beetles is tested. Beetles that fly freely for more than 10 s were selected as research subjects. In the experiment, the beetle was first placed in a sealed plastic bottle filled with carbon dioxide gas for about two and a half minutes. Then, the beetle of the coma state was taken out, and the beetle was fixed on the support by a string. Next, two electrical stimulation channels were drawn from the electronic backpack, which were connected to the left and right dorsal longitudinal muscle of beetle with an insect needle through a tetrafluoroethylene insulated silver wire, respectively. Finally, the electronic backpack was glued to the front backboard of the beetle with double-sided tape and beeswax. The beetle is suspended with a string to facilitate the beetle to start flapping. Figure 5 shows a beetle carrying with an electronic backpack.

### 3.2. Electrical Stimulation Signals

**Figure 6:**
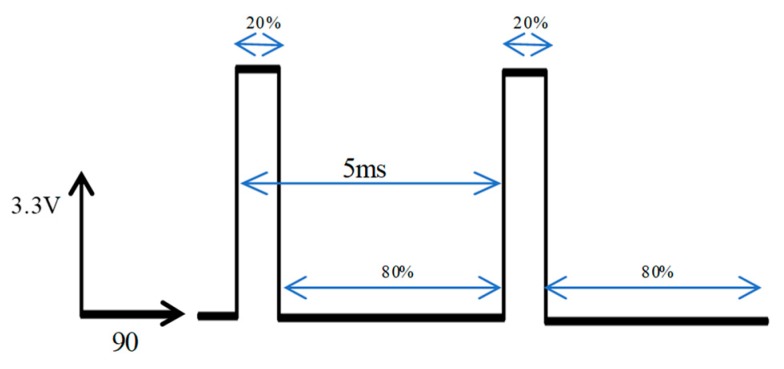
The stimulation signal of positive potential pulses sequence.

The PC controller interface on the computer can set the pulses number, period, duty cycle and stimulation channel of the stimulation instruction. The stimulation instruction is transformed into a specific stimulation signal through the electronic backpack, which stimulates the left and right dorsal longitudinal muscle of the beetle, controls the vibration of the beetle’s wings and records the response time of the beetle. The response time refers to the period between receiving electrical stimulation and the moment the beetle’s wings start to vibrate. The response time is a parameter that measures the response speed of the beetle and the effect of the flapping onset control after receiving electrical stimulation. When the response time decreases, it means that the wings flapping onset responds faster and the control effect becomes better. As an important evaluation parameter of beetle flight behavior, the response time still plays an important role in our future research. For example, we want to design a feedback controller to control the beetle’s flight path, the response time will be one of the feedback messages in the control algorithm. The response time reflects the real-time effect of a stimulation signal on correcting beetle flight behavior and decided the timing, length and times of the stimulation we applied. Therefore, we can improve and optimize the control algorithm and improve the effectiveness of control according to the response time, so as to obtain the optimal and controllable trajectory. The stimulation signal used in the experiment is shown in Figure 6, which is a positive potential pulses sequence (amplitude = 3.3 V, period = 5 ms, duty cycle = 20%, pulse number = 90).

### 3.3. Validation Experiment

**Figure 7:**
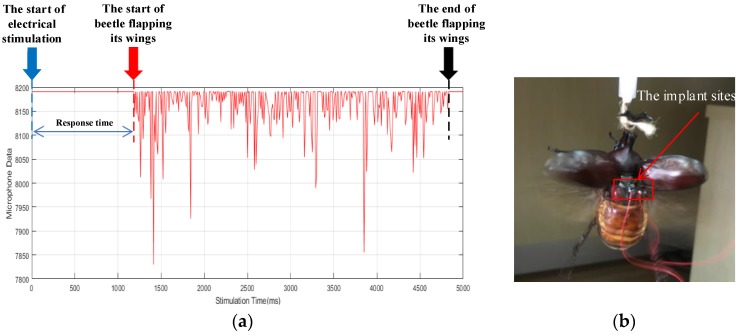

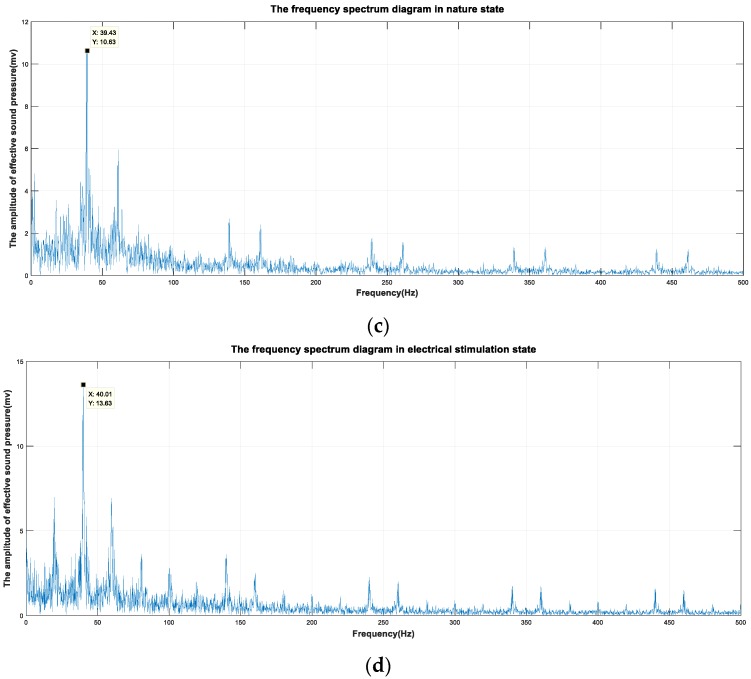
The time-domain and frequency-domain analysis of the wings flapping. (**a**) The time-domain diagram of the wings vibration signals in electrical stimulation state (the blue arrow indicates the beginning of the electrical stimulation, and red and black arrows indicate the start and end of beetle flapping its wings respectively); (**b**) the photograph of wings flapping; (**c**,**d**) the frequency spectrum diagrams of the wings vibration signals in nature state and electrical stimulation state.

After the experimental preparation, the stimulation instruction is delivered by the PC controller. The instruction is transmitted to the electronic backpack through the wireless network when the coordinator received the stimulation instruction through serial port. According to the instruction, the electronic backpack applied 90 positive potential pulses with a period of 5 ms and duty ratio of 20% in the pulse amplitude of 3.3 V to the left and right dorsal longitudinal muscle of beetle. The microphone is used on the electronic backpack (the sampling frequency is 1000) to collect the data of beetle wings vibration in electrical stimulation state and natural state. We drew the time-domain diagram of the wings vibration signals in the electrical stimulation state, as shown in Figure 7a. Then, we processed the collected data through digital signal processing, and drew the frequency spectrum diagrams of the wings vibration signals in the state of electrical stimulation and nature, as shown in Figure 7c,d. According to the frequency spectrum diagrams, the wings vibration frequency is 39.43 Hz in natural state and 40.01 Hz in the electrical stimulation state. Considering the measurement error, the surrounding environment and other influencing factors, they are approximately the same although there are some differences in the vibration frequency of the wings in the two states. Therefore, we infer that the electrical stimulation mode of wings vibration is consistent with the natural mode.

**Figure 8:**
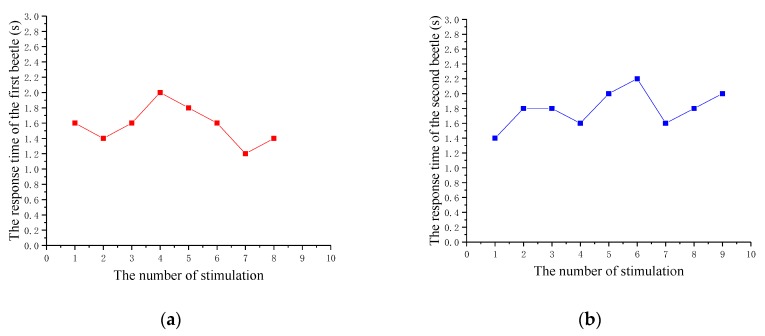
The response time of two beetles to each stimulation experiment. (**a**) The response time of the first beetle; (**b**) the response time of the second beetle.

In order to obtain an accurate response time, two beetles are selected in 10 experiments. The collected data of wings vibration are shown in Table 1 and graphed in Figure 8. According to the recorded data, the average response time of ten experiments per beetle and the average flapping onset success rate and average response time of two beetles were calculated. Figure 8 shows that the response time of beetles is about 2 s, which is basically the same. The average response time of two beetles is 1.69 s, which is less than 2 s. It can be seen that beetles can start flapping their wings in a short period of time. In the experiment, we find that when the electrical stimulation starts, the beetle’s elytron (the protective shell of the wings, which the beetle opens first in-flight) will open, but quickly retract until the wings vibrate. The natural flapping behavior of beetles is an autonomous behavior, while the flapping behavior of beetles in the electrical stimulation state is a passive behavior. Therefore, unlike autonomous behavior, electrical stimulation would lead to certain inhibitory behavior of beetle, so that the response time could not reach the milliseconds level, which is the typical time scale of insects’ natural behavior. These results show that the stimulation of the dorsal longitudinal muscle of beetles by electrical pulses can cause the wings of beetles to vibrate, and it is repeatable.

### 3.4. Comparative Experiment

Different electrical pulses parameters may have different influences on electrical stimulation effects. Different from the fixed parameters in the validation experiment, a series of comparative experiments are designed to study the effect of different parameters of the electrical pulses on wings vibration, including the number, the period and duty ratio of pulses. According to the verification experiment, a series of stimulation parameters that cause the beetle to flap the wings with a high probability are proposed. Only one stimulation parameter was modified each time, while the other parameters keep unchanged. The studied parameters were increased (decreased) from an initial value according to a certain interval until the beetle starts flapping the wings. We recorded the response time of the beetle in each stimulation and analyzes the sampled experimental data. Finally, an optimal stimulation parameter can be derived from the above series of experiments.

(1) The number of pulses

**Figure 9:**
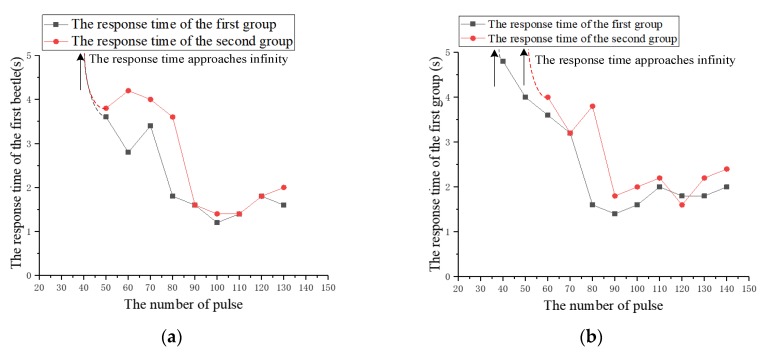
The relationship between the response time of beetles and the number of pulses. (**a**) The first beetle; (**b**) the second beetle.

**Figure 10:**
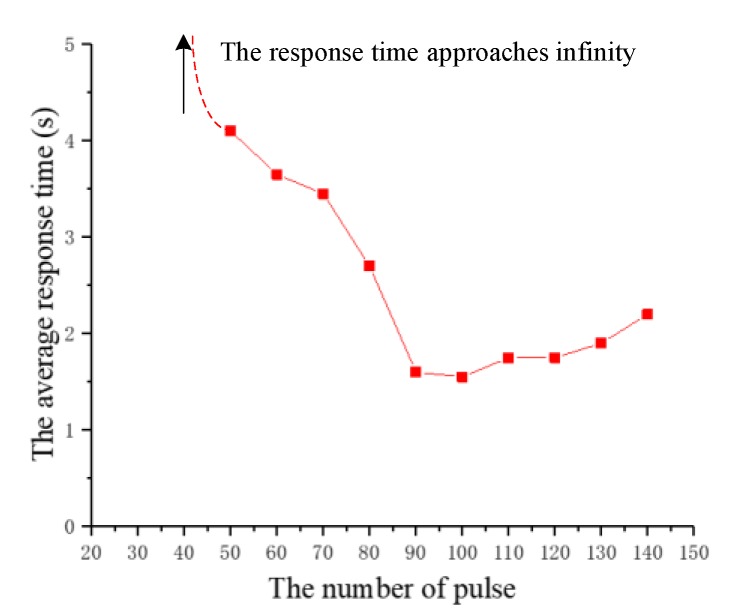
The relationship diagram between the average response time and the number of pulses.

The experiment studied the effect of the number of pulses on the response time. The number of pulses is increased from 20 to 150 at intervals of 10 and the other three parameters remain fixed. The number of pulses that successfully cause the beetle to flap the wings for the first time in the experiment is called the threshold of pulses number (an experiment with a response time of less than 5 s is considered successful). On the basis of this threshold, the number of pulses continues to increase by ten at a time, and the response time of five to eight experiments is recorded. This is a group of experiments repeated twice, as shown in Figure 9. According to the recorded data, the average response time of the two beetles is calculated and plotted as a graph, as shown in Figure 10.

During the experiment, we find that, prior to reaching the threshold, the abdomen of the beetle will produce certain wiggle or the wings will spread out. However, beyond that threshold, the beetle flaps the wings in a short time. Figure 9 shows the response time of two groups of beetles. Although there are some differences in the data of two beetles, the general trend is similar. Figure 10 illustrates the relationship between the average response time and the number of pulses. The experimental results indicate that an average threshold of the pulse number is about 50. The beetle can flap the wings rapidly only when the threshold is exceeded. Furthermore, after the threshold is exceeded, the response time of the beetle has a significant decrease tendency firstly, then increases slightly as the number of pulses increases. The response time of the beetle is the shortest when the number of pulses is 100. That is to say, the increase of the stimulation pulses number helps to reduce flapping onset time in the early stages. When the optimal stimulation pulses number is reached, the flapping onset time is extended if the number of pulses is further increased.

In view of this phenomenon, we speculate that the energy generated by electrical stimulation is in the stage of energy accumulation before reaching the threshold. When the threshold is reached, that is, when the energy accumulates to a certain value, it will cause the beetle to flutter its wings. After exceeding the threshold, increasing the number of pulses will further increase energy, and the increase of energy in the early stage can continue to shorten the flapping onset time of beetles, but when the number of pulses reaches the optimal value, the energy has increased to the limit value, and the beetle is in the fatigue period. If the number of pulses continues to be increased at this time, it will extend the response time.

(2) The period of pulses

**Figure 11:**
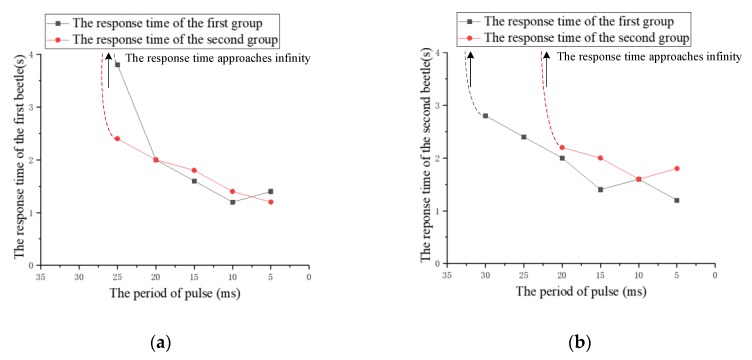
The relationship between the response time of beetles and the period of pulses. (**a**) The first beetle; (**b**) the second beetle.

**Figure 12:**
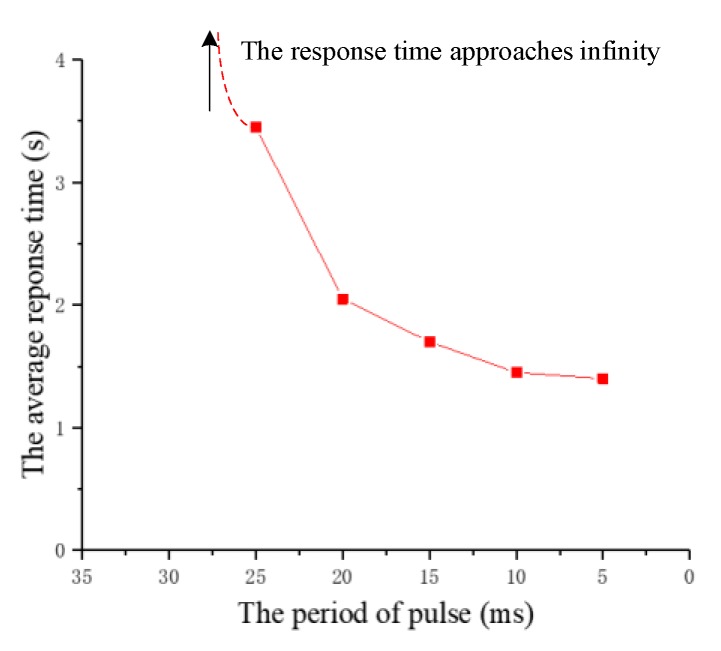
The relationship diagram between the average response time and the period of pulses.

The experiment studies the effect of the period of pulses on the response time. The period of pulses is decreased from 40 ms to 5 ms at intervals of 5 ms and the other three parameters remain fixed. Similarly, the period of pulses that successfully cause the beetle to flap the wings for the first time is called the period threshold in the experiment. On the basis of the threshold, the period of pulses continues to decrease by 5 ms at a time, and the response time of five to eight experiments is recorded. The relationship between the response time and the period of pulses for two beetles is shown in Figure 11. According to the recorded data, the average response time of the two beetles is calculated and plotted as a graph, as shown in Figure 12.

Although there are minor differences, the response time of the two beetles has a similar trend due to the increase of pulses period. The experimental results indicate that an average threshold of the pulses period is about 25 ms. It can be seen that the response time is determined by the pulses period after reaching the threshold. The response time is positively correlated with the pulses period. That is, we can reduce the flapping onset time of the beetle by the decrease of the stimulation pulses period. Additionally, the beetle has the shortest response time of 1.2 s when the period of pulses is 5 ms. For this law, we suppose that with the decrease of pulses period, the denser the energy produced by electrical stimulation, the shorter the flapping onset time of beetles.

(3) Duty ratio of pulses

**Figure 13:**
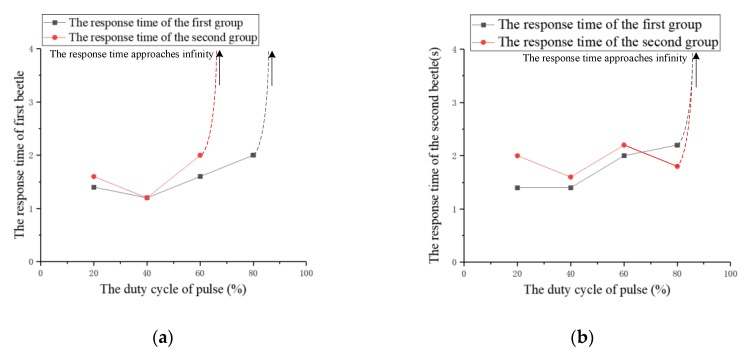
The relationship between the response time of beetles and the duty ratio. (**a**) The first beetle; (**b**) the second beetle.

**Figure 14:**
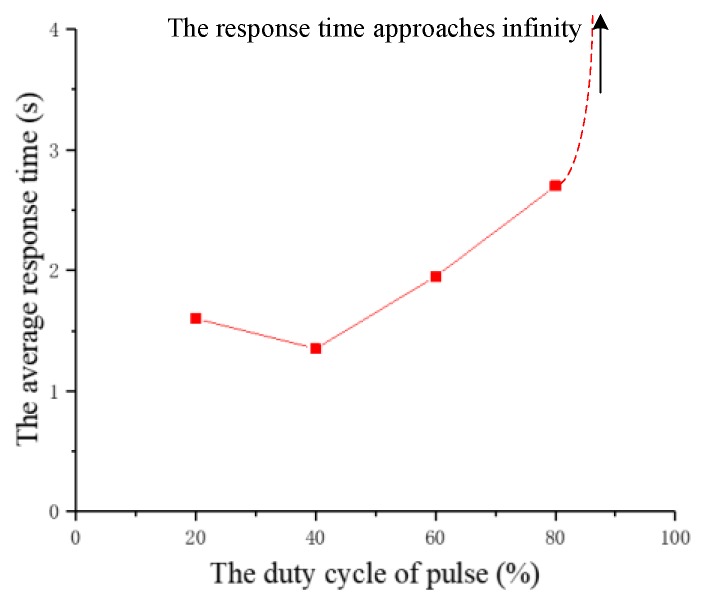
The relationship diagram between the average response time and the duty ratio.

The experiment studies the effect of the duty ratio of pulses on the response time. The duty ratio of pulses is increased from 20% to 100% at intervals of 20% and the other three parameters remain fixed. The duty ratio of pulses that successfully cause the beetle to flap the wings for the last time is called the pulses duty ratio threshold in the experiment. On the basis of this threshold, the duty ratio of pulses continues to increase from 20% to 100%, and the response time of each experiment is recorded. The relationship between the response time and the pulses duty ratio for two beetles is shown in Figure 13. According to the recorded data, the average response time of the two beetles is calculated and plotted as a graph, as shown in Figure 14.

Although there are minor differences, the response time of the two beetles has a similar trend due to the increase of the duty ratio. The experimental results indicate that the average threshold of the duty ratio is about 80%. The response time decreases at first and then increases with the increase of the duty ratio. The experimental results demonstrate that the increase of the duty ratio within a certain range can improve the control effect of wings flapping onset. However, the response time becomes longer when the duty ratio is further increased. After the duty ratio exceeds 80%, we find that the beetle’s wings will not flap and the abdomen produces a violent twist. Furthermore, when the experiment was finished, we find that the beetle’s flying ability became weaker and the beetle lost a certain degree of vitality. Therefore, we infer that the explanation that the higher duty ratio of the electrical pulses induces too large the stimulation intensity for the beetle, which has generated a certain amount of damage to the beetle. Additionally, the response time has the shortest value of 1.2 s when the duty cycle of pulses is 40%.

According to the analysis of the experimental data, the comparative experimental results illustrate the duty ratio of 40% for 100 cycles in the pulses period of 5 ms and amplitude of 3.3 V is the optimum stimulation parameter.

## 4. Conclusions

In this paper, a beetle flapping onset control method based on electrical stimulation is proposed. The left and right dorsal longitudinal muscles are selected as stimulation sites to control the flapping onset behavior. Firstly, we expound the principle of beetle flapping onset control based on electrical stimulation and analyzes the movement function of the dorsal longitudinal muscle for the flapping onset control of the beetle. Then a wireless micro-control system, which includes the PC controller, the coordinator, and the electronic backpack, is designed to control beetle flapping onset. The PC controller is used for delivering the stimulation instructions and receiving and processing flight information of beetles. The coordinator communicates with the PC controller through a serial port protocol and serves as a data channel for the PC controller and the electronic backpack. The electronic backpack is carried on the beetles and communicates with the coordinator through the wireless communication protocol for executing stimulation instructions and collecting and transmitting vibration information of the wings. Finally, verification and comparison experiments are implemented. The validation experiment verifies the effectiveness of dorsal longitudinal muscle stimulation with respect to the beetle flapping onset. The validation experimental results illustrate that when the period, duty ratio, number of and amplitude of pulses stimulation signal are assigned to 5 ms, 20%, 90 and 3.3 V respectively, the beetle flapping onset can be controlled with an average response time of 1.69 s. In order to obtain the optimal stimulation parameters and shorten the response time, the comparative experiments are performed. In the comparative experiments, we selected the optimal stimulation parameters by studying the effects of three different pulses parameters on wings flapping effect, including the number, the period and duty ratio of pulses, which is conducive to our later stage more efficient control of beetle flight behavior. The comparative experimental results illustrate that the optimization of duty ratio from 20% to 40%, and the number of pulses from 90 to 100, are proved to the best parameter configuration.

## Figures and Tables

**Table 1:** Sampling data of two beetles for 10 experiments.

The Number of Experiments	The Response Time of the First Beetle (s)	The Response Time of the Second Beetle (s)
1	1.6	1.4
2	1.4	1.8
3	1.6	1.8
4	-	1.6
5	2	2
6	1.8	-
7	-	2.2
8	1.6	1.6
9	1.2	1.8
10	1.4	2
The average response time	1.58	1.8

“-” represents electrical stimulation failed to control the wings vibration.
